# What were they thinking when providing preference measurements for generic health states? The evidence for HUI3

**DOI:** 10.1186/s12955-018-0993-9

**Published:** 2018-08-15

**Authors:** David Feeny, William Furlong, George W. Torrance

**Affiliations:** 1 Department of Economics and Centre for Health Economics and Policy Analysis, McMaster University, Hamilton, ON Canada; 2 Health Utilities Incorporated, Dundas, ON Canada; 3 Centre for Health Economics and Policy Analysis, McMaster University, Hamilton, ON Canada; 4 McMaster University, Hamilton, ON Canada; 5 Department of Economics, McMaster University, Kenneth Taylor Hall 426, 1280 Main Street West, Hamilton, ON L8S 4M4 Canada

**Keywords:** Chance Board, Feeling Thermometer, Health-related quality of life, Health Utilities Index, Validity, Multi-attribute utility, Preference measurement, Preference scores, Standard gamble, Quality-adjusted life years, Attributes, Impacts

## Abstract

**Background:**

Multi-attribute generic preference-based measures of health-related quality of life are used as comprehensive outcome measures. Typically preferences for health states defined by these systems are elicited from a representative sample of the general population. An important element in that elicitation process is the information that respondents were instructed to consider in providing their responses.

**Methods:**

A random sample of community-dwelling respondents in Canada was surveyed in face-to-face interviews. Respondents provided preference scores for selected Health Utilities Index Mark 3 (HUI3) health states. Respondents also answered questions about the most important attributes and the importance of various impacts of the health states in providing their preference scores.

**Results:**

Fifty per cent of respondents reported that they focussed on two, and 21% on three, attributes of the eight HUI3 attributes. Each of the eight attributes was identified as important; pain (49%), vision (37%), cognition (34%), emotion (28%), and ambulation (28%) were the most important. The null hypothesis that all of the attributes were equally important was rejected (*p* < 0.001). With respect to the impacts, 89% of respondents indicated that the ability to take care of oneself was quite or very important; similarly 76% reported the same for impact on family life, 69% for impact on the happiness of others, 61% for the impact on their ability to work, and 42% for the impact on their leisure activities. The null hypothesis that all of the impacts were equally important was rejected (*p* < 0.001).

**Conclusions:**

In providing preference scores for HUI3 health states, respondents thoughtfully examined the implications of the health states for their ability to live, work, socialize, and function.

## Introduction

Generic multi-attribute preference-based measures of health-related quality of life (HRQL) are widely used as comprehensive outcome measures in clinical studies, including economic evaluations and health technology assessments of healthcare, and in population health surveys. By convention, scores of these measures are on a scale in which dead = 0.00 and perfect health = 1.00; therefore these measures can be used to integrate mortality and morbidity as is done in calculating summary measures of health such as quality-adjusted life years (QALYs). Prominent examples of these measures include the EQ-5D-3 L [21], the Health Utilities Index Mark 2 (HUI2) [28], the Health Utilities Index Mark 3 (HUI3) [12], the Quality of Well Being Scale (QWB) [16], and the Short-Form 6D (SF-6D) [1].

Each of these multi-attribute systems has a health-status classification system that describes a large number of health states and a multi-attribute utility function that provides a preference-based score for each of those health states. There is substantial evidence on the validity of the health-status classification systems and the multi-attribute utility function scoring systems for these measures [6].

However, relatively little information is reported in the literature on what respondents who provided the preference scores used to estimate these multi-attribute utility functions were instructed to consider. Key to the appropriate interpretation and use of any multi-attribute preference-based scores is an understanding of how the scores were elicited and what factors respondents considered in providing their valuations. This paper provides such information for HUI3.

The importance of such information is highlighted in guidelines for reporting standards for utility assessment [24] and multi-attribute utility-based instruments [29]. Stalmeier and colleagues specify 25 categories (many with sub-categories) of information that should be reported, as applicable. Relevant with respect to the theme of this paper is C12 (p 204): “What was the subject instructed to assume, if anything, regarding costs to him or her or family about the possible outcomes?” Xie and colleagues in discussing reporting guidelines on scoring functions for multi-attribute preference-based measures list 21 items classified in seven sections. Of relevance here is Item 11 (p 872): “Mode of Data Collection is Stated.” More generally, McDowell and Newell [18] note that “we know relatively little about the process of making subjective health judgements”.

An example of a report on the conduct of a preference elicitation survey that reports on the experiences of respondents is Devlin et al. [2]. Devlin and colleagues summarize the written comments provided by respondents to an EQ-5D-3 L postal survey in New Zealand and conclude that “the valuation exercise imposes a substantial cognitive burden on respondents and many do not understand it” (p 1265).

An example of an important reason for knowing what factors respondents were instructed to consider is the controversy about the extent to which the productivity consequences of health states are reflected in preference scores. The original Panel on Cost Effectiveness in Health and Medicine assumed that the effects of morbidity on productivity were incorporated in the preference scores and therefore the monetary (pecuniary) consequences need not be estimated separately ([13], pp. 181–182). The Panel argued that including the monetary estimates would result in double counting. Several subsequent studies have examined the extent to which the effects of health on productivity in the labor market and household production are reflected in the preference scores, casting doubt on the original Panel’s position [17, 23, 27]. Sculpher and O’Brien [22] report that respondents in the Measurement and Valuation of Health study (used to estimate the scoring UK function for the EQ-5D-3 L; see [3]) were given no explicit guidance on this issue. The Second Panel on Cost Effectiveness in Health and Medicine concluded that it is unlikely that productivity effects are indeed captured in the utility scores and instead recommends that these effects be captured in monetary terms [19]. This is an example of why what respondents were or were not instructed to consider is important information for interpreting the scores.

The aims of this paper include reporting on the factors respondents were instructed to consider, and did consider, in providing preference scores used to estimate a multiplicative multi-attribute utility function for the HUI3 system [4, 11]. Another aim is a recommendation that the current reporting guidelines be expanded to include a description of what factors respondents were instructed to consider and whether or not respondents were debriefed about what they were thinking while providing their preference scores. This paper is based on information derived from the debriefing of respondents in the HUI3 scoring project. The results of the debriefing have implications for the content and construct validity of the HUI3 system. The paper reports previously unreported results from a broader study. The relevant methods from the broader study are described briefly in the Background section, followed by a description of the methods, results, discussion, and conclusions for the specific topics upon which this paper focuses.

## Background

**Fig. 1:**
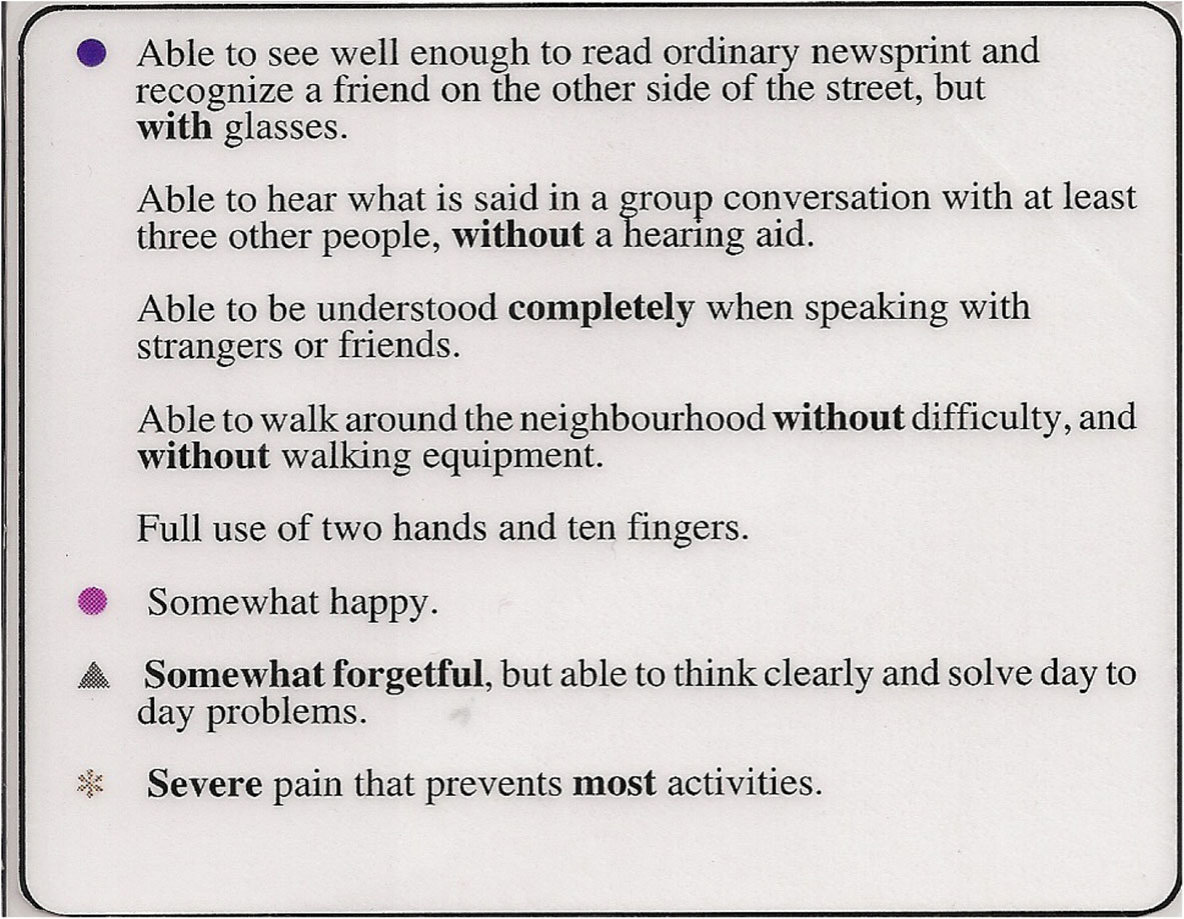
Example of an HUI3 Health-State Description Card. Legend of symbols for attribute levels: no symbol – attribute at level 1 (no disability); circle – level 2; triangle – level 3; square – level 4; diamond (not shown) – level 5 if attribute has 6 levels; asterisk – lowest functional level, level 5 if attribute has 5 levels or level 6 if the attribute has 6 levels. Note. A health-state description consists of one level for each of the eight HUI3 attributes: vision, hearing, speech, ambulation, dexterity, emotion, cognition, and pain and discomfort. In vector notation, the health state above is written as V2,H1,S1,A1,D1,E2,C3,P5

HUI3 consists of two major components: a health-status classification system; and a preference-based scoring function for valuing the health states described by that system. The health-status classification system describes an individual’s health status at a point in time based on eight attributes (or dimensions or domains) of health. The attributes in conventional order are: vision (V), hearing (H), speech (S), ambulation (A), dexterity (D), emotion (E), cognition (C) and pain and discomfort (P). There are five or six levels per attribute, varying from normal to severe disability. For example, pain varies from “free of pain and discomfort” to “severe pain that prevents most activities”. (The full HUI3 health-status classification system is shown in the Appendix.) An individual’s comprehensive health state is described as an eight-element vector, one level for each attribute; an example is provided in Fig. 1.

The second major component is the scoring function that provides utility scores on the conventional dead to perfect health scale for all of the health states defined by the health-status classification system. The scoring function is based on directly measured preference scores obtained from a random sample of the general population survey respondents (community preferences). The procedures for eliciting the preference scores from the respondents are described briefly below. More comprehensive descriptions are found in Furlong et al. [11] and Feeny et al. [4].

Respondents were asked to score a series of HUI3 health states. Respondents were told that they would live in each of the various HUI3 health states described for the rest of their life, which was explicitly defined as the duration of each respondent’s self-nominated additional life expectancy in years. The interview script explicitly instructed respondents to focus only on the health-state descriptions and to keep all other factors constant: “When imagining yourself in these health states please remember that where you live, your income, your friends, and family would be the same as now.”

The HUI3 preference measurement survey included two widely used preference elicitation instruments: a visual analog scale known as the Feeling Thermometer (FT) and the standard gamble implemented using the Chance Board (CB). (The FT is presented as Fig. 1 in [10], p 82; the CB as Fig. 2, p 83. http://www.chepa.org/Files/Working%20Papers/WP%2090-9.pdf). Respondents were asked to rank order a set of HUI3 health states on the FT in which the top of the scale was labelled “Most Desirable” and the bottom of the scale was labelled “Least Desirable”. Figure 1 is an example of how health-state descriptions were presented to respondents. In particular, note the use of symbols to assist respondents in identifying the attributes for which there was any disability and the extent of that disability. The number of health states which a respondent was asked to evaluate on the FT varied from 14 to 18 depending on the survey group to which the respondent was randomized. A sub-set of four to nine states (depending on the survey group to which the respondent was randomized) were subsequently measured using the CB. With the CB the respondent was offered a lottery, Choice A, consisting of perfect health state (all attributes at level 1, no disability) with probability p and the least preferable health state (either the all-worst health state (all attributes at their lowest level, highest degree of disability) or being dead, depending on the CB question) with probability 1- p versus Choice B, an intermediately ranked state, with probability *p* = 1.00 (i.e., for sure). Probability p in Choice A is varied systematically until the subject is indifferent between the Choice A and Choice B. The more desirable the health state in Choice B, the higher the indifference probability.

After completing the FT and CB tasks, respondents were asked nine demographic questions. Respondents were then asked 11 debriefing questions, each with multiple-choice or open-ended response options about their opinions regarding the importance of health-status attributes and impact factors in the preference measurement interview process. Assessments of debriefing questions having quantifiable responses are the focus of this paper. Note that the debriefing questions were asked after the questions that elicited scores for the HUI3 health states presented to that respondent.

The preference-elicitation survey instrument for the HUI3 scoring function project involved detailed word-by-word interview scripting of instructions to respondents, use of well-tested props, in-person interviewing, intensive training of professional interviewers and provision of a custom interviewer manual, pre-testing and practice interviews, and audio-recording of all interviews for quality assurance. The survey instructions focussed on health-state descriptions, the context for the preference measurement of health states (for instance, the instruction that “your income, your friends, and family would be the same as now”), and the preference measurement techniques that respondents were to use for the health states presented to them. The survey instrument was designed to minimize the consideration of extraneous factors and minimize intra- and inter-interviewer effects. All these factors are important design components of the preference elicitation survey that should be reported so that users of the resulting scoring function can make informed judgments about the methodologic rigour and quality of the foundational preference measurements.

## Methods

This paper focuses on the respondent debriefing questions with quantifiable response options asking respondents about the importance of the health attributes and impact factors they were thinking about when responding to the preference measurement questions (Table 1). The preference measurement interview structure was predicated on the assumption that respondents have well developed but not readily articulable preferences for health states and, therefore they need an opportunity to think and reflect before providing their response. The interview process was structured to give the respondent ample opportunity and a structured set of tasks to help them contemplate, discover, and state their preferences. Thus respondents were asked to describe their own health by identifying the most appropriate level for each attribute of the HUI3 health-status classification system, and the rank ordering and rating using the FT was followed by further intense reflection by having to make choices on the CB.

**Table 1:** Respondent Interview Debriefing Questions^a^

Did one or two of the health state characteristics figure more importantly in your decisions? If so, which ones?	
How important were the following in your decisions to place, or choose, the health states? I will read out a list, and for each one I would like you to tell me if they were not at all important, slightly important, somewhat important, quite important, or very important.	
The effect it would have on your leisure activities?	
The effect it would have on your family life?	
The effect it would have on your ability to work at your current job?	
The effect it would have on your ability to look after yourself?	
The effect it would have on the happiness of others?	
As a result of the interview, have any of your opinions, or the strength of your opinions and feelings changed about the different types of health we have discussed?	

^a^Questions in order as presented to respondents (*n* = 504)

Preference measurement scores for the HUI3 health states were obtained in a survey of a random sample of the English-speaking general population (age 16 years and older) in the city of Hamilton, Ontario, Canada. One debriefing question asked if respondents focussed on one or two health-state characteristics (attributes). We investigate whether all attributes are considered important, and test the null hypothesis that all attributes are equally important to preference measurement at the group level. We also compare the importance ratings of the attributes to the prevalence of disability in those attributes using data on the prevalence of any disability among the respondents (*n* = 504) and in the general population (from the [25], *n* = 11,924).

Another question asked respondents about the importance of five potential impacts on activities of living in their scoring of the health states. We measured the importance of each of the five factors and test the null hypotheses that each of the five factors is important and that the five factors are of equal importance.

The minimum level of statistical significance is *p* < 0.05. The minimum size for difference in rates regarded as important is 5 %. Chi-square tests were used to test differences in rates (i.e., proportions).

## Results

The demographic characteristics of the sample (*n* = 504 respondents) are presented in Table 2. Respondents varied in age from 16 to 85 with a mean of 43 years. Nearly 60% were female and most reported good or better health. The frequency distributions of the demographic characteristics for the sample of respondents are very similar to the census distributions reported by Statistics Canada for the general community-dwelling population [4, 11].

**Table 2:** Socio-Demographic Characteristics of Respondents (*n* = 504)

Demographic Variable	Variable Category	% of Respondents
Age (years):: mean = 43 years (SD 18)		
Gender	Female	59
	Male	42
Present health rating	Excellent	27
	Very good	42
	Good	22
	Fair	8
	Poor	2
Marital status	Single	29
	Married/common-law	52
	Divorced/Separated	12
	Widowed	7
Education	High school or less	50
	Apprenticeship, certificate, part or complete Bachelor’ degree	45
	Post-graduate or Professional degree	5
Religion	Roman Catholic	32
	Anglican	14
	United	13
	Other Protestant	11
	Other, Non-Protestant	17
	No religious preference	14
Employment status	Employed	56
	Unemployed	4
	Unable to work due to health problem	6
	Retired/Student/Keeping house	31
	Other	3
Family income^a^	Low (< 70% of national median^a^)	38
	Middle (70% to 165% of national median)	43
	High (> 165% of national median)	15
	Refused	4

Note: Category percentages may not sum to 100& due to rounding

^a^The categories for Family Income variable categories relative to median for Canada during survey year

Source: Statistics Canada. Annual Estimates for Census Families and Individuals (T1 Family File). http://www23.statcan.gc.ca/imdb-bmdi/document/4105_D5_T1_V10-eng.htm (accessed August 26, 2016)

The professional interviewers reported that 74.4% of respondents were extremely cooperative; for 76.2% of respondents the degree of thought was a great deal or quite a bit; and for 75.8% the overall quality of the interviews was very good or good ([4], pp. 122–123).

When asked if there were particular health attributes upon which the respondent focussed, there was considerable variation among respondents and attributes (Table 3). Each of the attributes was reported as being of particular importance in making preference measurement decisions by 5% or more of respondents. The reporting rates of particular importance varied substantially among attributes (*p* < 0.001) and there were important size (> 5%) differences in rates among attributes. For example the rate for the most frequently reported attribute of particular importance (pain at 49%) was approximately seven times the rate for the attribute least frequently reported (speech at 6.9%). Furthermore, most respondents reported multiple attributes of particular importance: 50% of respondents reported two attributes and 21% of respondents reported three attributes. Six per cent of respondents said that none of the attributes were more important than the rest.

**Table 3:** Attribute Importance and Rates of Disability by Attribute

HUI3 Preference Survey Respondents		General Population
Attribute Importance Rate (*n* = 504) %	Attribute Disability Rate (*n* = 504) %	Attribute Disability Rate (*n* = 11,924)* %
Pain (49.0)	Vision (62.1)	Vision (50)
Vision (36.9)	Pain (49.0)	Cognition (26)
Cognition (33.9)	Cognition (28.8)	Emotion (21)
Ambulation (28.0)	Emotion (25.2)	Pain (20)
Emotion (28.0)	Ambulation (12.3)	Hearing (5)
Hearing (17.1)	Speech (9.5)	Ambulation (3)
Dexterity (17.1)	Hearing (8.5)	Dexterity (2)
Speech (6.9)	Dexterity (8.3)	Speech (1)
*p* < 0.001.	p < 0.001.	
Intra-survey importance vs disability, *p* < 0.001.		
Inter-survey importance vs disability, *p* < 0.001.		

Note: Column percentages do not sum to 100 because attributes assessed independently

Legend: p = *p*-value; * Statistics Canada. Housing, Family and Social Statistics Division. General Social Survey Analysis Series. Health Status of Canadians: Report of the 1991 General Social Survey. Catalogue No. 11-612E, No. 8. ISBN 0–660-15392E, No. 8. Ottawa, Canada 1994

Table 3 reports the frequency of any disability for each attribute as reported by respondents to the preference measurement survey (Column 2) and in a large general population health survey. Attribute importance rates of preference survey respondents (Column 1) differed significantly (*p* < 0.001) from both the disability rates of the preference survey respondents (Column 2) and disability rates of the general population health survey respondents (Column 3). While vision had the highest rate of disability, pain was rated as the most important attribute. Further, while 28% rated ambulation as particularly important, the rates of disability in ambulation were 12.3% among preference survey respondents and 3% in the general population. Pain, vision, and cognition were the three attributes most frequently reported to be particularly important and among the four attributes with the highest prevalence of disability in both the preference survey respondent and the general population samples.

Each of the five impact factors was considered at least somewhat important (*p* < 0.003) by 70% or more of respondents when making FT and CB preference measurement decisions (Table 4). Some impact factors were reported as being important more frequently than others (*p* < 0.001). The impact factor most frequently reported to be important was the ability to look after oneself (96%). The least frequently reported was leisure activities (71%).

**Table 4:** Importance of Impact Factors: Ranking from Most to Least Importance

Life Impact Factor	Frequency of Respondents Reporting (%)				
	Not at all or Slightly Important	Somewhat Important	Quite or Very Important	Total	*p*-value
Looking after self	20 (4)	35 (7)	449 (89)	504 (100)	< 0.0001
Family life	66 (13)	55 (11)	383 (76)	504 (100)	< 0.0001
Happiness of others	70 (14)	86 (17)	348 (69)	504 (100)	< 0.0001
Working current job	116 (23)	86 (17)	302 (60)	504 (100)	< 0.0001
Leisure activities	146 (29)	146 (29)	212 (42)	504 (100)	0.0002
All (sum)	418 (16.6)	408 (16.2)	1694 (67.2)	2520 (100)	
*p*-value	< 0.001				

Note: Column percentages may not sum to 100 because the row factors were assessed individually

Finally, when asked if their opinions had changed during the interview process, most respondents (83%) reported that their opinions or strength of their opinions and feelings had not changed at a result of the interview.

## Discussion

The preference measurement interviews involved numerous challenging tasks. On the Feeling Thermometer, respondents are instructed to rank health states on a vertical visual analogue scale and ensure that the distance between states on the scale reflected the relative strength of their preference differences among states. On the Chance Board respondents were asked to compare their preference for health state in Choice B to the their preference for the probabilities of perfect health and, in general, the all-worst state in Choice A.

As noted above, the professional interviewers considered more than 70% of the respondents to be cooperative and thoughtful and rated the quality of interviews as high. Respondents understood and followed instructions. When asked about the tasks involved in the interview, respondents frequently accurately summarized the instructions that they were given. Further, 83% of respondents reported that the measurement process did not change their opinions or feelings about various health states. This result is consistent with our a priori hypotheses that people have well developed preferences for health states that can be measured in well designed and executed face-to-face interviews.

More than 98% of respondents reported some attributes to be more important than others, but as a group respondents focussed on all of the HUI3 health attributes, including both physical and mental health attributes. Each of the eight attributes was reported as being important by a substantial (> 5%) proportion of respondents. A possible limitation of the debriefing is that asking if one or two attributes were more important, we may have encouraged respondents to provide a positive response thereby exaggerating the effect at the individual level but illuminating the variability in the importance of attributes at the group level. More generally, a limitation of the study is that how we asked the questions may have influenced the responses provided by the respondents.

That each of the eight attributes was regarded as important is evidence consistent with the content validity of the HUI3 health-status classification system and multi-attribute utility function. Further the lack of correspondence between the prevalence of disability by attributes and the importance attached to the attributes implies that the importance of attributes should not be assumed to be based on the degree of familiarity with the disability associated with that attribute, that is by the prevalence of disability for that attribute.

The results also show that respondents considered the impacts of disabilities in the health states described. As a group they reported considering a wide variety of impacts of the health states as important, including: their ability to look after themselves, their family life, the happiness of others, their ability to work at their current job and the effects on their leisure activities. The five impact factors listed reflect recommendations about the health impacts that should be reflected in preference scores ([13], p 109).

Three of the factors listed focussed on the impacts of the health state for the respondent (ability to look after self; ability to work; leisure activities); two focussed on impacts on others (family life; happiness of others). An implication is that their preference scores reflect consequences both for self and for others. This in turn has implications for the interpretation of scores and the issue of accounting for spillover effects in cost-utility analyses [7, 20, 26].

Gold et al. [13] argue that role function should be included in health-status classification systems. The “within the skin” approach embodied in the HUI3 has been criticized because social role function is not explicitly included in the health-status classification system [5]. However, respondents to the preference survey appear to have taken social roles into account when providing preference scores. That the happiness of others and family life were regarded as important factors might imply that respondents did take the effects of health states on social interaction into account in providing scores.

Related evidence on this issue is found in Hays et al. [14] who report results from a linear equating algorithm to map responses from PROMIS Global Health Items to overall HUI3 scores. The PROMIS scales used in the analyses included physical functioning, depressive symptoms, pain interference, ability to participate in social roles and activities, anxiety, and pain rating. In the results reported by Hays and colleagues the PROMIS domains that were predictive of overall HUI3 scores and included in the mapping algorithm are physical functioning, pain interference, anxiety, depressive symptoms, and ability to participate in social roles and activities. It is noteworthy that although social activities are not an HUI3 attribute, the ability to participate in social roles and activities is an important predictor of overall HUI3 scores. One interpretation of this result is that social activities in HUI3 are distal, not proximal, such that deficits in HUI3 attributes (proximal effects, for instance, of disabilities in hearing or cognition) imply restrictions on the ability to participate in social roles (distal) that influence the score for the HUI3 health state — that respondents took these consequences into account in providing the scores for health states. Further, there is overlap between the PROMIS domains that predict HUI3 scores and the attributes and factors that were regarded as important by the respondents, providing evidence of the construct validity of the HUI3 system.

With respect to the potential double-counting productivity issues discussed in the Introduction, a majority of respondents indicated that they took their ability to work at their current job into account in providing preference scores for health states. This does not necessarily imply that respondents ignored the interview script instructions that their income would be the same regardless of the health state. That the majority of respondents took the ability to work into account could imply that people value the capacity to engage in meaningful activities and does not necessarily imply that respondents are incorporating the monetary effects of labor force participation in the utility scores they provide. The extent to which respondents took the monetary effects of work into account, as opposed to the health-related quality of life effects, is unknown.

In general, the results underscore the importance of the substantial variability in preferences for health states and, typically, little of this variability is systematically associated with socio-demographic characteristics [9]. Respondents differed with respect to which attributes were the most important and which impact factors were most important, emphasizing the need for broadly representative samples of respondents. The results of the statistical tests indicate that while there is heterogeneity in preferences, not all attributes or all impacts are of equal importance. Responses to the questions indicate that respondents thought carefully about the implications of the health states for their ability to engage in life.

The results are also consistent with a related theme, Kaplan’s Ziggy Theorem [15]. Kaplan argues that health is valuable because it provides the capacity to perform activities that are valued. Good health enables one to participate in valued activities.

Further, the results are consistent with observations by Dr. James Fries in discussing the fears of growing old expressed by patients. Dr. Fries notes that individuals dread pain, fear senility and the loss of memory, and fear being dependent on others ([8], p 411). Indeed these observations resonate with the views expressed by the HUI3 respondents.

This study provides important new evidence from a random sample of the general population about the factors that respondents consider in making preference judgments about health states. Collectively, respondents considered all eight HUI3 attributes to be important in defining health status (content validity), although there is considerable variability among individuals about which specific attributes are the most important. Similarly, there was heterogeneity in the impact factors considered to be the most important. The item with the highest level of importance was the ability to look after oneself. Finally including questions about what factors were important to respondents in providing their preference responses provides important information that is useful in interpreting preference scores for HUI3 health states.

Two issues relevant to all, or virtually all, generic preference-based measure raised by an anonymous reviewer (thank you) warrant discussion. The reviewer notes that the preference scores used to estimate multi-attribute utility functions are typically drawn from random samples of the general population and thus, for the most part, are provided by relatively healthy individuals. It is of course possible that those with more experience with ill health would rate health states differently than would a group of mainly healthy respondents. To the best of our knowledge, this potential limitation applies to all of the major generic preference-based measures. The issue warrants further research.

The reviewer also wonders if the values attached to health states change over time. Of course, the nature of our study design, a cross-sectional survey at a point in time, provides no evidence on the stability or lack of stability in preferences for health states over time. Again this potential limitation applies to all of the generic preference-based measures and has implications for the research agenda.

## Conclusions

People have well developed preferences for HUI3 health states that can be measured in well designed and executed face-to-face interviews. All HUI3 attributes are important. Attribute-specific disability rates should not be considered as a substitute for attribute importance ratings. Social interaction effects were taken into account by respondents when providing preference scores for HUI3 “within-the-skin” health states.

The explicit description of instructions given to respondents about what to, and what not to, consider in providing their preference scores for health states is important information. An implication is that information about the instructions should be incorporated into standard reporting practices for utility assessment studies in general, and studies reporting on the estimation of multi-attribute utility functions in particular. Similarly, the description of the process of any debriefing and reporting of the results from the debriefing also add value and should also be incorporated into reporting standards. Finally, we encourage further research, including formal qualitative and quantitative research on the role of the instructions given to respondents.

## Appendix

**Table 5:** Health Status Classification System: Health Utilities Index Mark 3 (HUI3)

Attribute	Level	Description
VISION	1	Able to see well enough to read ordinary newsprint and recognize a friend on the other side of the street, without glasses or contact lenses.
	2	Able to see well enough to read ordinary newsprint and recognize a friend on the other side of the street, but with glasses.
	3	Able to read ordinary newsprint with or without glasses but unable to recognize a friend on the other side of the street, even with glasses.
	4	Able to recognize a friend on the other side of the street with or without glasses but unable to read ordinary newsprint, even with glasses.
	5	Unable to read ordinary newsprint and unable to recognize a friend on the other side of the street, even with glasses.
	6	Unable to see at all.
HEARING	1	Able to hear what is said in a group conversation with at least three other people, without a hearing aid.
	2	Able to hear what is said in a conversation with one other person in a quiet room without a hearing aid, but requires a hearing aid to hear what is said in a group conversation with at least three other people.
	3	Able to hear what is said in a conversation with one other person in a quiet room with a hearing aid, and able to hear what is said in a group conversation with at least three other people, with a hearing aid.
	4	Able to hear what is said in a conversation with one other person in a quiet room, without a hearing aid, but unable to hear what is said in a group conversation with at least three other people even with a hearing aid.
	5	Able to hear what is said in a conversation with one other person in a quiet room with a hearing aid, but unable to hear what is said in a group conversation with at least three other people even with a hearing aid.
	6	Unable to hear at all.
SPEECH	1	Able to be understood completely when speaking with strangers or friends.
	2	Able to be understood partially when speaking with strangers but able to be understood completely when speaking with people who know me well.
	3	Able to be understood partially when speaking with strangers or people who know me well.
	4	Unable to be understood when speaking with strangers but able to be understood partially by people who know me well.
	5	Unable to be understood when speaking to other people (or unable to speak at all).
AMBULATION	1	Able to walk around the neighbourhood without difficulty, and without walking equipment.
	2	Able to walk around the neighbourhood with difficulty; but does not require walking equipment or the help of another person.
	3	Able to walk around the neighbourhood with walking equipment, but without the help of another person.
	4	Able to walk only short distances with walking equipment, and requires a wheelchair to get around the neighbourhood.
	5	Unable to walk alone, even with walking equipment. Able to walk short distances with the help of another person, and requires a wheelchair to get around the neighbourhood.
	6	Cannot walk at all.
DEXTERITY	1	Full use of two hands and ten fingers.
	2	Limitations in the use of hands or fingers, but does not require special tools or help of another person.
	3	Limitations in the use of hands or fingers, is independent with use of special tools (does not require the help of another person).
	4	Limitations in the use of hands or fingers, requires the help of another person for some tasks (not independent even with use of special tools).
	5	Limitations in use of hands or fingers, requires the help of another person for most tasks (not independent even with use of special tools).
	6	Limitations in use of hands or fingers, requires the help of another person for all tasks (not independent even with use of special tools).
EMOTION	1	Happy and interested in life.
	2	Somewhat happy.
	3	Somewhat unhappy.
	4	Very unhappy.
	5	So unhappy that life is not worthwhile.
COGNITION	1	Able to remember most things, think clearly and solve day to day problems.
	2	Able to remember most things, but have a little difficulty when trying to think and solve day to day problems.
	3	Somewhat forgetful, but able to think clearly and solve day to day problems.
	4	Somewhat forgetful, and have a little difficulty when trying to think or solve day to day problems.
	5	Very forgetful, and have great difficulty when trying to think or solve day to day problems.
	6	Unable to remember anything at all, and unable to think or solve day to day problems.
PAIN	1	Free of pain and discomfort.
	2	Mild to moderate pain that prevents no activities.
	3	Moderate pain that prevents a few activities.
	4	Moderate to severe pain that prevents some activities.
	5	Severe pain that prevents most activities.

The above level descriptions are worded here exactly as they were presented to interview subjects in the HUI3 preference survey

## Abbreviations

CB: Chance Board
FT: Feeling Thermometer
HRQL: Health-related quality of life
HUI2: Health Utilities Index Mark 2
HUI3: Health Utilities Index Mark 3
QALYs: Quality-adjusted life years
QWB: Quality of Well Being Scale
SF-6D: Short-Form 6D
